# EXPLORING FRAILTY PREVALENCE AND RISK FACTORS AMONG STROKE PATIENTS: A MULTICENTER CROSS-SECTIONAL STUDY

**DOI:** 10.1093/geroni/igae098.1774

**Published:** 2024-12-31

**Authors:** Haiyan He, Siyuan Tang, Wenting Peng, Kehan Liu, Shuomin Wang, Yaru Bai, Chongmei Huang, Minhui Liu

**Affiliations:** Central South University, Changsha, Hunan, China (People’s Republic); Central South University, Changsha, Hunan, China (People’s Republic); University of Washington, Seattle, Washington, United States; Central South University, Changsha, Hunan, China (People’s Republic); Central South University, Changsha, Hunan, China (People’s Republic); Ningxia Medical University, Yinchuan, Ningxia, China (People’s Republic); Ningxia Medical University, Yinchuan, Ningxia, China (People’s Republic); Ningxia Medical University, Yinchuan, Ningxia, China

## Abstract

Frailty is prevalent among stroke patients, and it is strongly associated with higher risks of expected adverse outcomes. However, the risk factors for frailty remained unclear in middle-aged and older patients with stroke. We aimed to identify the prevalence and risk factors of frailty in middle-aged and older patients with stroke in Chinese hospitals. A cross-sectional survey was employed using a random sampling method, involving middle-aged and older patients with stroke in two tertiary hospitals in Central and Northwest China between January 2022 and June 2022. Frailty was assessed with the Frail Scale. Candidate predictors included nine socio-demographic factors and 12 clinical factors. We used generalized linear models to examine the relationship between frailty and related factors. Among 838 patients, 560 were male and 278 were female, with the majority aged between 50-60 years (30.4%) and 60-70 years (27.6%). The prevalence of pre-frailty and frailty was 40.1% (n=338) and 31.1% (n=261), respectively. We found that the stroke type (β = -0.30, 95% CI: -0.46, -0.14; P < 0.001), abnormal nutritional risk (β = 0.23, 95% CI: 0.01, 0.45; P = 0.042), hyperlipidemia (β = 0.21, 95% CI: 0.03, 0.39; P = 0.026), and post-stroke disability (β = 0.59, 95% CI: 0.53, 0.64; P < 0.001) were significantly associated with frailty scores in stroke patients. Our finding provides evidence regarding the frailty status of stroke patients. It suggests that early screening and intervention for these factors are crucial to preventing or improving frailty among middle-aged and older patients with stroke.

